# Molecular determinants of antimicrobial resistance in *Klebsiella pneumoniae* isolates among geriatric patients in Chattogram, Bangladesh: a cross-sectional study

**DOI:** 10.1128/spectrum.00145-26

**Published:** 2026-07-16

**Authors:** Mahmud Hassan Arif, Afroza Akter Tanni, Farhana Yasmin, Mirza Nurul Karim, Enshad Ekram Ullah, Md. Abdus Sattar, Jannatul Ferdous, Nazia Farha, Anjasu Paul, Sadman Yousuf Sajid, Nahid Sultana, S. M. Rafiqul Islam, Md. Mahbub Hasan, Adnan Mannan

**Affiliations:** 1 Department of Medicine, Chittagong Medical College 467859, Chattogram, Bangladesh; 2 Department of Genetic Engineering & Biotechnology, Faculty of Biological Sciences, University of Chittagong 54493, Chattogram, Bangladesh; 3 Next-generation Sequencing, Research and Innovation Laboratory Chittagong (NRICh), Biotechnology Research and Innovation Centre (BRIC), University of Chittagong 54493, Chattogram, Bangladesh; 4 Department of Microbiology, Chattogram Maa O Shishu Hospital 247357, Chattogram, Bangladesh; Tel Aviv Sourasky Medical Center, Tel Aviv, Israel

**Keywords:** *Klebsiella pneumoniae*, geriatric patients, multidrug resistance, whole genome sequencing, chattogram, sequence types

## Abstract

*Klebsiella pneumoniae* (KPN) infections pose heightened risks in the geriatric population due to weakened immunity, prevalent comorbidities, potential exposure in long-term care settings, and increased likelihood of antibiotic resistance (ABR). The study focused on the prevalence and antibiotic resistance of KPN infections, the presence of ABR genes in KPN, and the genomic characterization of KPN obtained from geriatric patients in Chattogram. A total of 543 specimens were collected from four hospitals in Chattogram, along with demographic data from hospital records. Genomic DNA was extracted from multi-drug-resistant (MDR) KPN, and the presence of ABR genes*, bla_TEM-1_*, *sul-1*, *aadB*, *bla_NDM-1_*, *bla_SHV-11_*, and *phoE* was identified. To characterize the KPN genomes, two MDR KPN isolates were subjected to whole-genome sequencing (WGS), and the data were analyzed using bioinformatics tools to identify genomic determinants of ABR. KPN exhibited high resistance to ceftazidime (96%), cefuroxime (92%), and cefixime (83%), but sensitivity to colistin (79%) and amikacin (75%). MDR KPN was mostly detected in sputum (36%) and urine (27%) specimens, where the prevalence of ABR genes, *blaTEM-1*, *sul1*, *aadB*, *blaNDM-1*, and *blaSHV-11* were 28.2%, 17%, 6.17%, 56%, and 48% of these strains, respectively. These genomes exhibited distinct profiles for sequence types, ST420 and ST277 in Kpn007 and Kpn016, respectively, and ABR genes (*qnrS1*, *blaCTX-M-15*, and *blaSHV-27*), virulence factors (*ybt*, *iuc1*, *iro1*), and contained both K (K20, K46) and O antigens (O1, O3b). MDR KPN in the geriatric population poses a serious health concern due to their increased vulnerability to infections and limited treatment options, requiring careful management.

**IMPORTANCE:**

Multidrug resistance (MDR) and the hypervirulence nature of *Klebsiella pneumoniae* (KPN) in geriatric patients pose a critical health concern in nosocomial infections worldwide and result in high clinical complexity and mortality. The study investigated the factors for KPN infections and analyzed antimicrobial resistance profiles. More than 60% of *Klebsiella pneumoniae* isolates from geriatric patients were resistant to third- and fourth-generation cephalosporins, and most isolates carried *bla_NDM-1_ and bla_SHV-11_* genes. Analyzing whole genomes of two KPNs, Kpn007 (ST277) was identified as a hypervirulent strain with aerobactin and yersinia siderophores, contributing to virulence, and Kpn016 (ST420) carried fluoroquinolone (*qnrS1*), ESBL (*bla_CTX-M-15_* and *bla_SHV-27_*) resistance. Both genomes contained K antigens (K20 and K46) and O antigens (O1 and O3b).

## INTRODUCTION

*Klebsiella pneumoniae* (KPN), the most medically relevant *Klebsiella* species, is responsible for a significant proportion of hospital-acquired urinary tract infections, pneumonia, septicemia, and soft-tissue infections, and is commonly found on hospital equipment (1). Additionally, KPN is much better suited to the hospital environment than other *Enterobacteriaceae* due to its superior ability to survive on hands and other surfaces and to spread quickly, causing nosocomial epidemics (2). KPN is mostly found in the gastrointestinal system, digestive system, and to a lesser extent in the nasopharynx in humans, where it can cause illness by entering the bloodstream or other organs (3). However, these colonization rates in hospitalized patients significantly rise in direct proportion to the number of days spent in the hospital and the use of antibiotics (4).

*Klebsiella* species can form colonies on mucosal surfaces without causing pathology. It is an opportunistic pathogen that causes hospital- and community-acquired infections, particularly among geriatric patients. Elderly patients have a weakened immune response and reduced functional reserve, making them more vulnerable to bacterial infections. Due to physiological changes, weakened immune responses, comorbidities, polypharmacy, and frequent hospitalizations, elderly individuals are highly susceptible to antibiotic-resistant *K. pneumoniae* infections (4, 5), which often lead to prolonged hospital stays, increased morbidity, and high mortality rates, making multi-drug-resistant (MDR) *K. pneumoniae* a serious public health concern. Elderly patients with carbapenem-resistant infections have significantly higher risks of adverse outcomes, including ICU care, respiratory failure, septic shock, and 90-day mortality (6).

Elderly patients infected with KPN in outdoor settings may face challenges in accessing timely medical care and may be at increased risk of exposure to environmental sources of the bacterium. In contrast, indoor elderly patients, particularly those residing in long-term care facilities or hospitals, may have a higher prevalence of KPN due to proximity to other infected individuals and the potential for transmission in healthcare settings, necessitating stringent infection control measures to mitigate spread and ensure effective treatment (6). While treatment options for elderly patients can be challenging to identify, the differences between them and younger individuals are minimal. One of these challenges is adjusting antibiotic dosing to account for age-related changes in pharmacodynamics and pharmacokinetics in the elderly (7). Drug absorption and compliance in the gastrointestinal tract, for example, are lower in patients with comorbidities (8). Additionally, comorbid patients typically take many drugs at the same time, emphasizing the importance of monitoring for potential drug interactions (9).

KPN exhibits high resistance to several antibiotics, including beta-lactams, fluoroquinolones, and aminoglycosides, and this resistance has been growing rapidly worldwide over the past several years due to excessive antibiotic use (10). The development of beta-lactamase enzymes that hydrolyze carbapenems is one of the main defense mechanisms used by *Enterobacteriaceae* against beta-lactam antibiotics (11). The primary mechanisms of resistance to aminoglycosides and fluoroquinolones include reduced uptake, poor drug absorption or accumulation in bacteria, and modification of enzymes that inactivate the antibiotic (12), especially mutations in the quinolone resistance-determining regions of the *gyrA* and *parC* genes (13). Moreover, cephalosporin resistance is frequently mediated by the production of β-lactamases, particularly extended-spectrum β-lactamases (ESBLs) and carbapenemases. Temoneria (TEM) and *Klebsiella pneumoniae* carbapenemase (KPC) enzymes are among the most clinically significant β-lactamases (14). TEM β-lactamases were initially narrow-spectrum but have evolved to hydrolyze extended-spectrum cephalosporins, reducing their efficacy (15). KPCs (class A carbapenemase) confer resistance to almost all β-lactam antibiotics, including carbapenems, which are often considered the last line of defense against MDR gram-negative bacterial infections. Furthermore, *sul-1* encoding KPN interferes with the folic acid synthesis pathway by synthesizing sulfonamide-resistant dihydropteroate synthase enzyme, and *aadB* encodes an aminoglycoside adenylyl transferase, an enzyme that inactivates specific aminoglycoside antibiotics. *NDM-1* is a class B group of metallo-beta-lactamase, and *SHV-11* is a class A group of extended-spectrum beta-lactamase that are a major concern because of showing resistance to beta-lactam antibiotics and extended-spectrum cephalosporins. The dissemination of these resistance genes, often carried on plasmids, contributes to the rapid spread of resistance within healthcare settings (16).

The genetic composition and diversity of KPN contribute to its capacity to develop AMR and hypervirulence. Based on previous studies, the KPN genome ranges from 5 to 6 Mbp, with 5,000–5,800 protein-coding genes, including approximately 1,700 essential genes, as well as an extensive list of auxiliary genes involved in virulence, antimicrobial resistance, and environmental adaptation (17). Extensive genomic research into the comprehensive features and mechanisms of *K. pneumoniae* has led to its classification into specific sequence types (STs). Based on multilocus sequence typing (MLST), these categories represent a diverse range of distinct lineages, including ST11, ST14, ST15, ST17, ST23, ST29, ST45, ST66, ST101, ST147, ST149, ST231, ST258, ST307, ST380, ST627, and ST977 (18, 19). In addition, ST420 belongs to the CG420 lineage and is predominantly associated with ompK36, whereas ST277 represents an exception in ompK36 grouping (20). Among them, ST11, ST15, ST132, and ST43 were mostly common in MDR KPN strains found in Bangladesh (21, 22).

The burden of AMR is rising in South Asian countries due to excessive and irrational use of antibiotics for treating community-acquired and hospital-acquired infections, where *E. coli*, *K. pneumoniae*, *S. aureus*, and *A. baumannii* are the leading antimicrobial-resistant microorganisms (ARMs) across all age groups (23–25). Furthermore, *K. pneumoniae*, *S. typhi,* and *E. coli* are the predominant ARMs in geriatric patients with pneumonia, acute respiratory infections, urinary tract infections, and sepsis (23). However, ciprofloxacin-resistant *S. typhi*/*S. paratyphi* were the prevalent ARMs in South and Southeast Asian countries in 2014, according to WHO reports (24). In addition, a large proportion of patients in Bangladesh with extended-spectrum cephalosporin-resistant Enterobacterales, and the prevalence of MDR *E. coli* and *K. pneumoniae,* showed difficulty in treating resistance phenotypes among community and hospital patients in 2019 (25, 26). In elderly patients with bacteremia in the ICU in Bangladesh, *Staphylococcus spp*., *E. coli,* and *K. pneumoniae* were highly resistant to ceftriaxone (72.13%) (5, 27).

This study aimed to investigate the prevalence and antibiotic susceptibility of KPN in Chattogram by analyzing clinical samples and demographic data of geriatric patients. This study also checked the prevalence of several resistance-associated determinants in KPN, *blaTEM-1, phoE*, *sul-1*, *blaNDM-1, blaSHV-11,* and *blaKPC*. Furthermore, whole-genome sequencing was used to identify genetic determinants associated with antimicrobial resistance in selected MDR *K. pneumoniae* strains from geriatric patients.

## MATERIALS AND METHODS

### Study setting and clinical data collection

This study was conducted at Chittagong Medical College Hospital, Chattogram Maa O Shishu Hospital, Chevron Clinical Laboratory (Pte.) Ltd., and Epic Health Care Ltd between April 2023 and May 2024. Clinical samples were collected from 543 geriatric patients in this molecular and epidemiological investigation. The study included samples from patients aged >60 years (according to the WHO guidelines) who were either outpatients or inpatients. Sampling was continued from clinical specimens, mainly including blood, urine, tracheal aspirate, throat swab, wound swab, sputum, and pus.

### Identification and selection of KPN strains

Clinical specimens were initially enriched in Luria-Bertani (HiMedia) broth and incubated at 37°C for 18 h. Following that, the samples were cultured on MacConkey agar media (HiMedia Laboratories, India) and incubated overnight at 37°C. Large, mucoid, and lactose-fermenting single colonies were selected for biochemical testing, including Triple Sugar Iron (TSI) and Simmons’s Citrate tests, which were performed to inferentially detect *Klebsiella pneumoniae* (KPN) (12), and the large, mucoid, and lactose-fermenting single colonies confirmed *Klebsiella pneumoniae* isolates were stored in skimmed milk at −20°C.

### Antibiotic susceptibility assay

Antibiotic susceptibility testing of the selected strains was performed using the Kirby-Bauer disc diffusion method. The susceptibility testing results were interpreted according to the criteria recommended by the Clinical and Laboratory Standards Institute (28). *K. pneumoniae* ATCC70603 was used as a quality control strain for antimicrobial susceptibility testing (29). Clinically used 13 antibiotics documented in the study were cotrimoxazole (25 µg), ciprofloxacin (5 µg), gentamicin (10 µg), ceftriaxone (25 µg), cefixime (5 µg), colistin (10 µg), amikacin (30 µg), cefuroxime (30 µg), tigecycline (15 µg), nitrofurantoin (300 µg), cefepime (30 µg), levofloxacin (5 µg), and ceftazidime (30µg).

### Screening of *phoE*, *bla_TEM-1_, sul1*, *aadB*, *bla_NDM-1_*, *bla_KPC_*, and *bla_SHV-11_* in KPN strains

Genomic DNA was extracted using the boiling method: large, mucoid, and lactose-fermenting single colonies were picked with a loop from bacterial cultures, suspended in 1,000 µL nuclease-free water, and centrifuged at 10,000 rpm for 10 min. After discarding the supernatant, the pellet was resuspended with nuclease-free water and heated at 95°C for 10 min, followed by a cold shock by immediately transferring it to −20°C for 30 min. The suspension was then centrifuged at 10,000 rpm for 10 min, and the supernatant containing DNA was collected and used as the PCR template. The plasmid DNA was extracted using Favorgen Plasmid DNA Extraction Mini Kit according to the manufacturer’s protocol (29, 30). Isolates were identified as *Klebsiella pneumoniae* through biochemical testing, and the presence of the *phoE* gene was confirmed in all isolates by PCR amplification (16). Multidrug-resistant genes, *bla_TEM-1_*, *sul1*, *aadB*, and *bla_NDM-1_* (31), in plasmid DNA and *bla_SHV-11_* in bacterial genomic DNA were detected using sequence-specific target primers (Table S1).

### Whole genome sequencing

Among all MDR isolates, the two MDR KPN isolates were randomly selected for whole-genome sequencing and processed for library preparation after genomic DNA extraction using a commercial DNA extraction kit (QIAamp DNA extraction kit, Qiagen, Germany) according to the manufacturer’s instructions. Immediately after this step, the DNA was subjected to library preparation using the NEBNext Ultra II FS DNA library prep kit (Illumina, San Diego, CA) following the manufacturer’s guidelines, and the library size was selected with AMPure XP beads (BECKMAN COULTER) (32). The final library was prepared and loaded on the Illumina iSeq100 by a paired-end 300-cycle protocol (33).

### Quality control, trimming, assembly, and annotation

Different software was used for analysis in default settings. FASTQC v.0.11.9 (34) and Trimmomatic v0.40 (35) were used for raw sequence quality checks and low-quality read and adapter region filtering, respectively. Then, SPAdes 3.14.1 was used for the *de novo* assembly, followed by QUAST v5.2, which was run for genome statistics (36, 37). The CBI Prokaryotic Genome Annotation Pipeline (PGAP) was run for annotation (38). Then, the taxonomy and identification of species and associated AMR-virulence factors were determined using Kraken2 and KLEBRATE (39, 40). The Rapid Annotation using Subsystems Technology (RAST) server V.2.0 was used to identify protein-coding and RNA genes (tRNA and rRNA) from assembled genomes, assign functions, predict subsystems, and build metabolic networks. The SEED analysis was done using the RAST database (41).

### Statistical analysis

Statistical analyses were conducted in GraphPad Prism 9. A Sankey plot was made using the networkD3 R package to visualize relationships in the data set. Antibiotic-resistant patterns (MDR, XDR, and DDR) were categorized, and the mean hospital stay and cost were determined using IBM SPSS Statistics Version 27 (IBM Corp., Armonk, NY, USA). Finally, R was used for linear regression to investigate correlations, and the ggplot2 package was used to present the results.

## RESULTS

### Demographic features of study populations

In the study, *Klebsiella spp*. infections were identified and cultured from clinical specimens of 543 geriatric patients at three hospitals in Chattogram. All samples were selected through a nonrandomized sampling procedure. All the patients in the entire study population were geriatric, with an average age of 71 years. Out of them, 57% were male, and 43% were female. Additionally, 44% of patients were hospitalized, while the remaining 56% were non-hospitalized.

Patients living in urban areas (61%) had a higher prevalence of *Klebsiella* infections than those from rural areas (39%) (Table 1). Clinical characteristics and admission history were analyzed. Most patients had respiratory tract infections (36%), followed by urinary tract infections (27%). Among patients for whom hospital stay length and patient type were available, we found that 56% of infections were community-acquired among outdoor patients, and the remaining 44% were hospital-acquired among indoor patients (Table 1).

**TABLE 1:** Demographic and clinical characteristics of *Klebsiella* infections in the study population

Variable	Prevalence (%) (*n* = 543)
Age	71.39 ± 7.54^*a*^
Gender	
Female	43%
Male	57%
Sample	
Blood	1.70%
Pus	9.40%
Sputum	36%
Throat swab	2.80%
Tracheal aspirate	18%
Urine	27%
Wound swab	5.00%
Patients type	
Indoor	44%
Outdoor	56%
Area	
Rural	39%
Urban	61%
Status	
Double-drug resistant (DDR)	25%
Multi-drug resistant (MDR)	29%
Single-drug resistant (SDR)	27%
Sensitive to all drugs	19%

^*a*^ Mean ± SD; %.

### Antibiotic susceptibility of *Klebsiella pneumonia*e isolates

**Fig 1:**
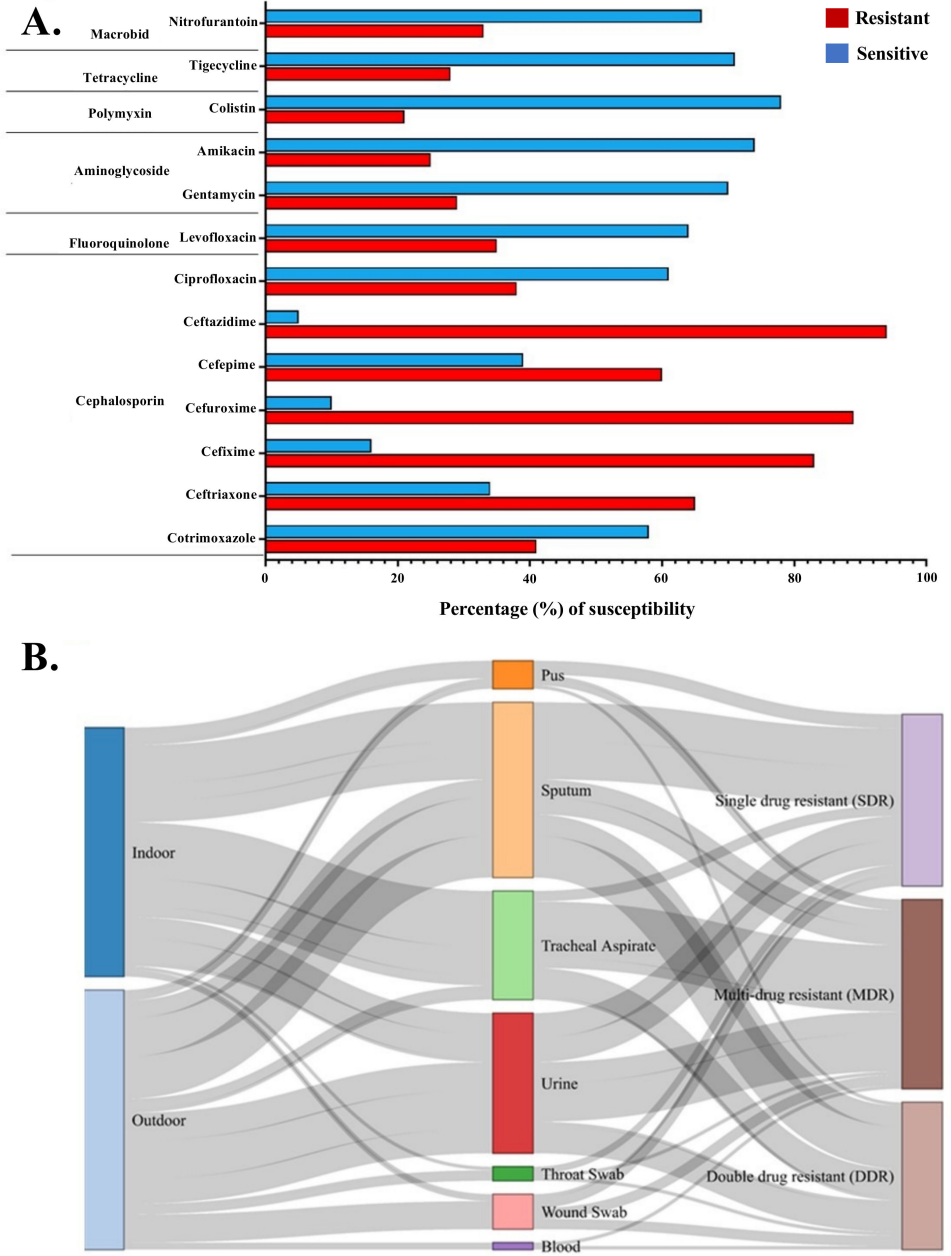
Antimicrobial susceptibility profiles and distribution of clinical specimens and resistance categories. (**A**) Antimicrobial susceptibility testing results for the 13 tested antibiotics by disc diffusion methods. (**B**) Sankey diagram visualizing the association between patient type (indoor/outdoor), clinical specimen source, and antibiotic resistance classification. The width of the bands represents the proportional volume of isolates.

Analyzing the proportion of *Klebsiella pneumoniae* isolates susceptible to each of the 13 tested antibiotics, we found that more than 60% of isolates were resistant to ceftazidime (92%), cefuroxime (89%), cefixime (82%), ceftriaxone (68%), and cefepime (61%) (Fig. 1A). On the other hand, more than 70% of the isolates were sensitive to amikacin, colistin, tigecycline, and gentamicin (Fig. 1A). Overall, 29% of KPN isolates were MDR, 25% were DDR, and 27% were SDR (Table 1). Among outpatients, *K. pneumoniae* was most prevalent in sputum (22.1%) and urine (18.2%). In contrast, the most common sources of infection among inpatients were tracheal aspirates (15.5%) and sputum (13.8%) (Fig. 1B). The Sankey plot shows that MDR isolates represented the most prevalent resistance category across nearly all sample sources. Sputum and tracheal aspirates served as the primary reservoirs for these resistant strains, contributing the highest volume of isolates to both the MDR and double-drug-resistant (DDR) pools. While pus and wound swabs contributed moderately to the MDR population, blood isolates, though fewer in number, were exclusively associated with MDR and SDR profiles (Fig. 1B).

**Fig 2:**
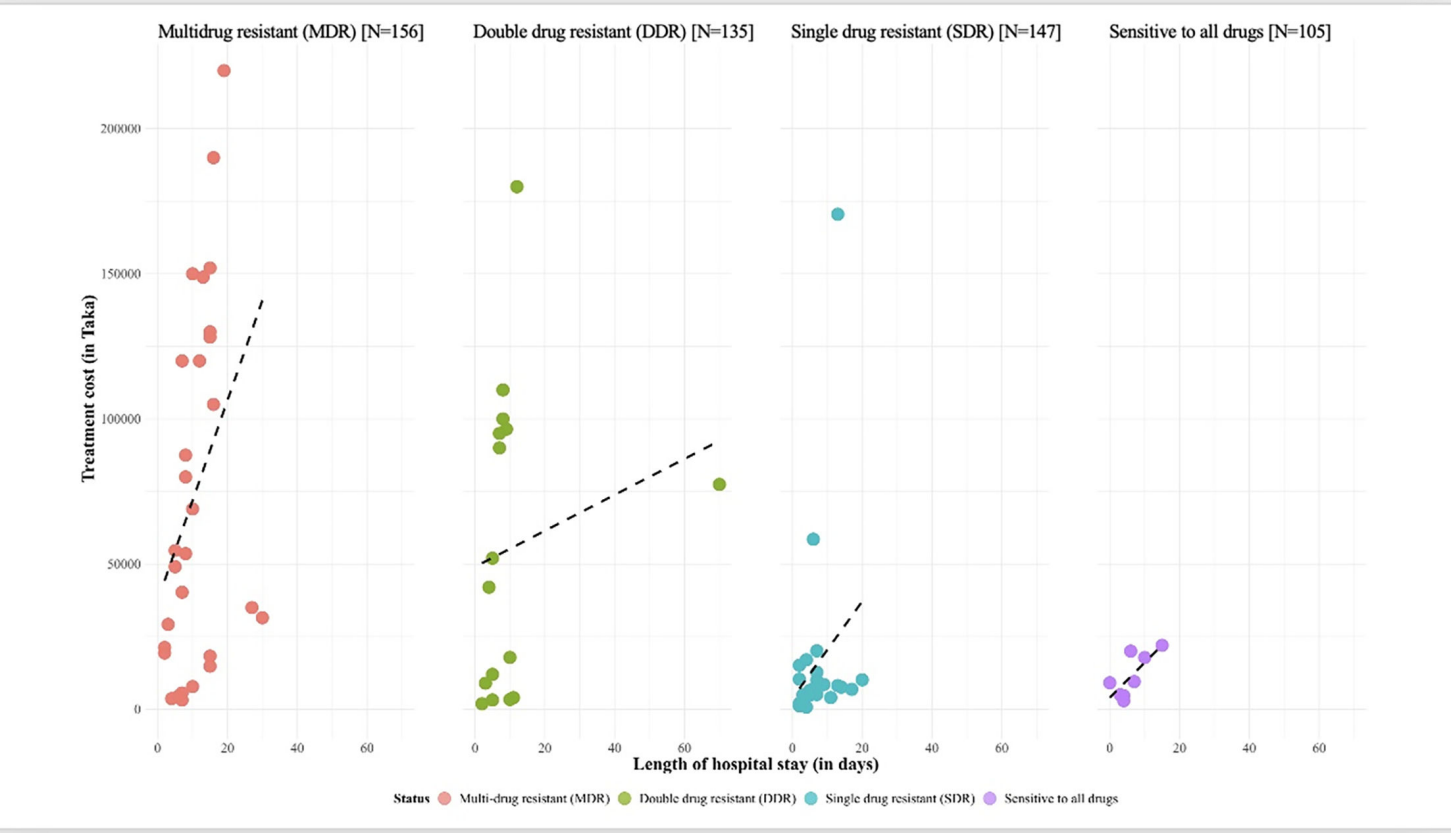
Correlation between treatment cost and length of hospital stay across different antibiotic resistance profiles.

Figure 2 shows a positive relationship between length of stay and treatment cost, more evident for MDR infections than for DDR infections. MDR patients have a steeper, more dispersed relationship between cost and stay, suggesting higher and more dispersed resource allocation, whereas for DDR infections, the relationship is moderate and narrow. In practice, MDR infections have a significantly higher financial burden. This can be justified by the fact that 28.7% of the admitted cases belonged to the MDR group, with a median length of hospital stay of 10 days and a median treatment cost of 478 USD, followed by DDR infections, which accounted for 24.9% of cases, with a median hospital stay of 6 days and a median treatment cost of 420 USD.

### Identification of *phoE*, *bla_TEM-_*_1_*, sul-1*, *aadB*, *bla_NDM-1_*, and *bla_KPC_* gene in KPN isolates

**Fig 3:**
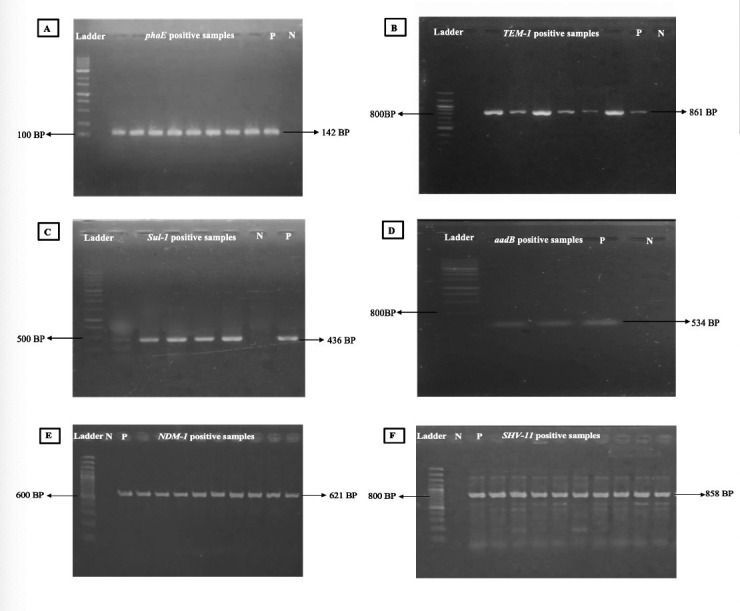
Molecular identification of the *phoE* gene and various antibiotic resistance determinants in *K. pneumoniae* isolates. Representative agarose gel electrophoresis images showing (**A**) *phoE* gene, (**B**) *bla_TEM-1_* gene, (**C**) *sul1* gene, (**D**) *aadB* gene, (**E**) *bla_NDM-1_* gene, and (**F**) *bla_SHV-11_* gene amplification. The product size of the *phoE* gene was 142 bp, and the product sizes of the other antibiotic resistance genes—*bla_TEM-1_*, *sul1*, *aadB*, *bla_NDM-1_*, and *bla_SHV-11_*—were 861, 436, 534, 621, and 858 bp, respectively. P denotes positive control, N for negative control, and a ladder was used as a reference to compare the bp of the target genes.

A total of 100 most resistant MDR KPN isolates were selected for molecular investigation, in which all samples contained the *phoE* gene, with a size of 142 bp on a 1.2% agarose gel (Fig. 3A), and these 100 isolates were screened for six related AMR genes, including *blaKPC*, *blaTEM-1*, *sul1, aadB*, *blaNDM-1*, and *blaSHV-11*. Of the isolates, 28.2% carried the *blaTEM-1* gene, as evidenced by an 861-bp band (Fig. 3). We did not find any *blaKPC*-positive KPN isolates in our study. Furthermore, 17% of the isolates were positive for the *sul1* gene, and *aadB* was detected in only 6.17% of isolates. Conversely, the *blaNDM-1* gene, confirmed at 631 bp on a 1.2% agarose gel, was detected in the highest proportion of isolates (56%), followed by *blaSHV-11* (48%) at 858 bp.

### Assembly statistics

After molecular screening, two MDR KPN isolates, Kpn007 and Kpn016, were selected for WGS sequencing from the sputum specimen, the most prevalent sample type in this study. The WGS analysis further confirmed that the isolates were *Klebsiella pneumoniae*. Analyzing the sequencing data, it was found that the estimated genome sizes are 5,442,638 and 5,565,244 bp for Kpn007 and Kpn016, assembled into 63 and 93 contigs, respectively, with GC contents of 57.2% and 57.1%, respectively. In addition, the number of protein-coding sequences within the genomes is about 5,137 and 5,280, with 71 and 79 non-coding RNAs (including tRNA and rRNA). The N50s of the genomes are 209,534 (Kpn007) and 240,835 bp (Kpn016), while the L50 is 8 for both genomes (Table S2).

### Gene prediction from KPN genomes

Phenotypic antimicrobial susceptibility testing of KPN isolates revealed that both strains have acquired resistance to a large proportion of clinically important antibiotics (Fig. 1A). On the other hand, several resistance genes were identified in the genomes of the Kpn007 and Kpn016 strains using the Kleborate tool, where the sequence types for Kpn007 and Kpn016 were found to be ST420 and ST277, respectively. The Kpn016 genome was identified as an MDR strain due to the presence of resistance genes *qnrS1*, *blaCTX-M-15*, and *blaSHV-27* on the chromosome, along with K46 and O3b, which are associated with virulence and disease defense in humans. On the other hand, the Kpn007 genome has K20 and O1 antigens identified as hypervirulent because of having the *ybt 9* gene in the genome, which is responsible for iron uptake, included in the yersiniabactin (Ybt) iron uptake system, and the *ybt* gene is most prevalent in the mobile genetic element, ICEKp3, found in KPN. In addition, the *iuc1* gene is responsible for aerobactin synthesis, along with the *iro1* gene, which encodes salmochelin siderophores found in hypervirulent KPN, which is responsible for iron acquisition in the bacterial genome. The *wzi95* (Kpn007) and wzi97 (Kpn016) genes found in both are responsible for membrane transporter synthesis (Table S2). Moreover, these genes were annotated and categorized into 573 subsystems via the RAST server (24).

**Fig 4:**
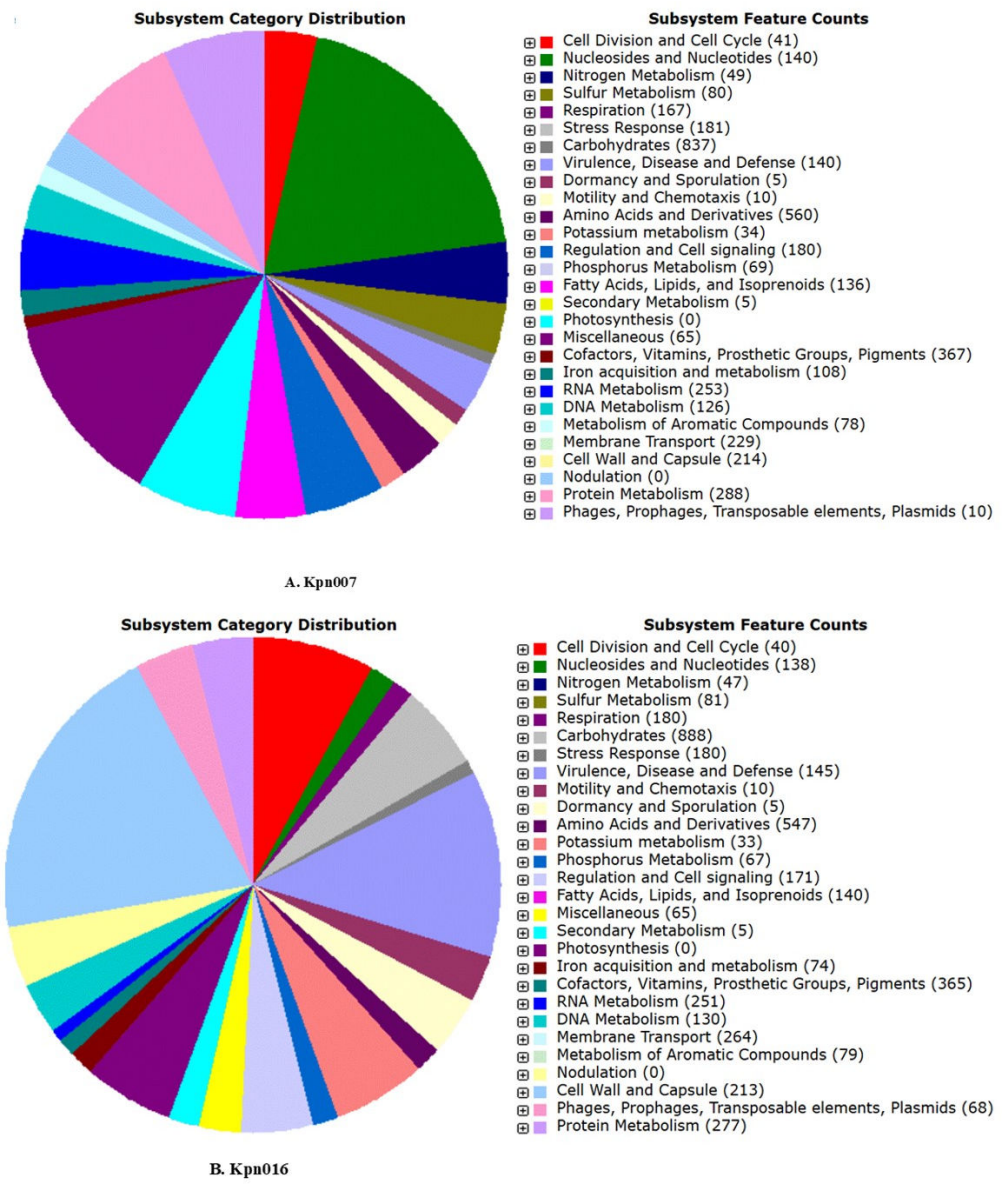
An overview of RAST subsystem category distribution annotation pie chart and subsystem feature count of annotated genes from the Kpn007 and Kpn016 genomes. Here, the number of genes for each category was represented in parentheses.

Most of the annotated genes found from both genomes were functioning in carbohydrates (837 in Kpn007; 888 in Kpn016), amino acids and derivatives (560; 547), cofactors, vitamins, prosthetic groups, and pigments (367; 365), protein metabolisms (288; 277), RNA metabolisms (253; 251), membrane transport (229; 264), cell wall and capsule (214; 213), and iron acquisition and metabolism (108; 74), which are crucial for bacterial survival and adaptation. In addition, both genomes carry 140 and 145 genes responsible for virulence, disease, and defense causing lethal effects in humans (Fig. 4 and Table S3).

## DISCUSSION

*Klebsiella pneumoniae* (KPN) is the second most prevalent cause of bloodstream infection in South Asia, including Bangladesh (42). The rise in multidrug resistance (MDR) amplifies the severity of morbidity and mortality associated with KPN infections in the elderly population. The investigation aimed to examine the frequency of KPN infections among geriatric patients in Chattogram, analyze antimicrobial resistance (AMR) patterns, and identify resistance genes and related virulence factors through whole-genome sequencing.

We reviewed epidemiological data for study patients to determine the age and sex distribution of KPN-infected individuals among hospitalized and outpatient patients. The demographic analysis revealed a higher susceptibility among males (57%). The higher incidence of KPN infection in males compared with females may vary due to a combination of behavioral, physiological, and immunological factors (43). These findings align with previous studies in Bangladesh, where the male patients are more likely to have higher exposure to healthcare facilities because of infections, underlying comorbidities like cardiovascular diseases, or elevated exposure to risk behaviors and repeated antibiotic use, contributing to the higher prevalence of antimicrobial resistance (25, 44). Patients living in urban areas (61%) had a higher prevalence of *Klebsiella* infection than those in rural areas (39%) due to increased urbanization and poverty. Furthermore, people are sharing their environments with urban terrestrial mammals, which are a common source of bacterial infection. It was found in one previous study that three serotypes of hypervirulent Kpn, for instance, K5, K20, and K57, were detected in community-acquired human infections, and these strains had potential transmitted sequence types of ST 23, ST35, and ST37 among them (45). Additionally, the transmission of mobile genetic elements in Kpn-positive isolates derived from urban people was highly prevalent compared to those from rural people in the farming sector, due to increased antibiotic use, self-medication, and non-prescribed antibiotic use, and human-created pollution at an accelerated rate (46). Furthermore, the higher population density and frequent hospital visits in urban settings may facilitate the transmission and spread of antimicrobial-resistant *K. pneumoniae* strains (47). In terms of overall frequency, KPN infections were most common in the intensive care unit (ICU), medicine wards, and adult surgery wards. Sputum (36%), followed by urine (27%) and tracheal aspirate (18%), was the most common source of infections (Table 1). Similar observations regarding specimen sources have also been identified in ICU units of the Surgery, Gynecology and Obstetrics, and geriatric sources of the hospital in China (48).

A similar study was conducted in Chattogram on patients across different age groups, in which KPN infections in urine (39.9%) were detected in patients under 15 years (49). Moreover, the infection rates from urine (18.2%) and sputum (22.1%) were observed among outdoor patients. On the other hand, tracheal aspirate was the source of infections among indoor patients (18.8%) (Fig. 1B). This may explain the high incidence of lung infections, cough, breathing difficulties, and urinary tract infections in the majority of patients, originating from the community or acquired in hospital settings. As a result, they are either admitted to the hospital for treatment or prescribed various medicines. Due to mismanagement or the dissemination of MDR bacterial strains in hospital settings, the treatment costs for patients infected with MDR KPN strains have been a global burden (50). In our study, patients with blood infections or bacteremia were uncommon in the study population. These results may have stemmed from the low usage of blood cultures in Bangladesh or from the potential use or administration of antibiotics prior to sample collection (51). KPN is a clinically important nosocomial pathogen that has developed resistance to antibiotics, particularly carbapenems, making KPN infections challenging to treat. The current study on KPN and its antibiotic resistance pattern shows that KPN isolates have developed resistance to second-generation and even third-generation cephalosporins, e.g., ceftazidime (92%), cefuroxime (89%), cefixime (82%), ceftriaxone (68%), and cefepime (61%), and are sensitive to colistin (82%), amikacin (78%), tigecycline (75%), and gentamicin (75%) (Fig. 1A). In a previous study, KPN isolates were resistant to cefepime, cefixime, and azithromycin and sensitive to amikacin, nitrofurantoin among neonates and adolescents (50). The study used molecular analysis to focus on KPN isolates and identified the *phoE* gene in all isolates. The investigation revealed the prevalence of beta-lactamase genes *blaTEM-1*, *blaNDM-1*, and *blaSHV-11*, indicating the intricate nature of these genes and their role in conferring multi-drug resistance. The study also revealed the *aadB* gene, conferring resistance to aminoglycosides, and the *sul-1* gene, conferring resistance to sulfonamides, which aligns with the previous studies conducted in Bangladesh. KPN encoding TEM-1, SHV variants, sul-1, and NDM-1 were found in clinical and environmental samples (canal water) and detected by whole-genome analysis and molecular experiments (52–54). Moreover, the samples studied tested negative for the blaKPC-2 gene. The gene encodes the KPC enzyme, which hydrolyzes carbapenems and causes hospital-acquired infections via plasmid-mediated horizontal gene transfer (55).

Analyzing whole genomes of KPN, we identified ST420 and ST277 in Kpn007 and Kpn016, respectively, with ST420 associated with multidrug-resistant KPN strains that contribute to the spread of antibiotic-resistant genes or transposable elements through horizontal gene transfer among bacterial populations, specifically targeting the carbapenem class of antibiotics (56). However, Kpn016, identified as ST277, is an MDR strain that carries the fluoroquinolone resistance gene qnrS1, which confers resistance to fluoroquinolones, e.g., ciprofloxacin and levofloxacin, and also harbors extended-spectrum beta-lactamase and virulence factors K46 and O3b (Table S2) (57). In addition, the strain carries the blaCTX-M-15 and blaSHV-27 genes, which confer resistance to beta-lactam antibiotics and contribute to the rapid global spread of high-risk clones such as ST11 in KPN (58). On the other hand, Kpn007 is a hypervirulent strain that carries the genes ybt9 and iuc1, which encode the synthesis of the siderophores yersiniabactin and aerobactin and the iron uptake systems (Table S2). Additionally, the maximum number of genes predicted in both genomes by the RAST server were involved in carbohydrates, amino acids and derivatives, cofactors, vitamins, prosthetic groups, cell wall and capsule synthesis, and iron uptake and acquisition systems, which are necessary for bacterial survival (Fig. 4).

According to our study (Fig. 2), MDR infections accounted for the highest proportion of cases (28.7%) and were associated with the longest hospital stay, expending a high amount of money (478 USD), indicating that the increase in treatment cost and duration of hospitalization is strongly associated with the degree of antimicrobial resistance, while SDR or DDR patients stayed for a shorter duration in the hospital with lower costs. These findings are consistent with studies showing that infections caused by MDR organisms, including MRSA, are associated with higher hospitalization costs and longer lengths of stay than those caused by susceptible organisms (59, 60). Similar observations have also been reported in systematic reviews, which showed that resistant infections incur additional treatment costs of several thousand US dollars and extended hospital stays (61, 62).

However, the study has some limitations. The sample size was limited, with isolates being collected from only four hospitals in Chattogram. A larger sample size would yield a larger dataset of genomic data, providing a more thorough understanding of bacterial genomes, AMR, and transmission patterns.

Implementing robust infection control strategies is critical to mitigating the dissemination of high-risk *K. pneumoniae* sequence types within the population. Future efforts should prioritize establishing a national antimicrobial resistance (AMR) surveillance network in Bangladesh to monitor longitudinal shifts in resistance genes and carbapenem susceptibility. Besides, future studies with large sample sizes are required to understand the devastating impact of antibiotic resistance nationwide and to take cautious measures in the use of antibiotics.

### Conclusion

This study investigated ESBL-producing *K. pneumoniae* isolates from geriatric patients in southern Bangladesh. The analysis allowed us to pinpoint the earliest occurrences of *blaTEM-1*, *blaNDM-1*, *blaSHV-11*, *sul-1*, and *aadB*, which encode β-lactamases and antibiotic resistance. The risk of bacterial infections among the geriatric population will increase significantly if specific *Klebsiella* strains resistant to antibiotics become more prevalent in the environment and hospital settings. This might intensify the persistence of the entire antibiotic resistance threat through horizontal and vertical gene transfer. To develop and implement plans to limit the abuse of antibiotics on a large scale and to provide alternative treatment options in cases of resistance, ESBL-producing organisms must undergo rigorous screening for *TEM-1*, *KPC*, and other antibiotic resistance genes (*SHV-11* and *NDM-1)* in Bangladeshi hospitals and community settings.

## Data Availability

The data used to support the findings of this study are included within the article. The Whole Genome Sequences of two KPN have been deposited in GenBank under the accession JBBMLU000000000.1 and JBBMLQ000000000.1. The Illumina Sequence reads are available in the NCBI Sequence Reads Archive (SRA) under accession numbers SRR25920011 and SRR25920015.

## SUPPLEMENTAL MATERIAL

Table S1 spectrum.00145-26-s0001.xlsx Primers and PCR conditions for the amplification of selected *K. pneumoniae* genes.

Table S2 spectrum.00145-26-s0002.xlsx Genomic information and Kleborate results of KPN.

Table S3 spectrum.00145-26-s0003.xlsx List of predicted genes and their functions_Kpn016 genome.

ASM does not own the copyrights to Supplemental Material that may be linked to, or accessed through, an article. The authors have granted ASM a non-exclusive, world-wide license to publish the Supplemental Material files. Please contact the corresponding author directly for reuse.

## References

[1] Choby JE, Howard-Anderson J, Weiss DS. 2020. Hypervirulent *Klebsiella pneumoniae* - clinical and molecular perspectives. J Intern Med 287:283–300. doi:10.1111/joim.13007 31677303 PMC7057273

[2] Barani M, Fathizadeh H, Arkaban H, Kalantar-Neyestanaki D, Akbarizadeh MR, Turki Jalil A, Akhavan-Sigari R. 2022. Recent advances in nanotechnology for the management of *Klebsiella pneumoniae*-related infections. Biosensors 12:1155. doi:10.3390/bios12121155 36551122 PMC9776335

[3] Chang D, Sharma L, Dela Cruz CS, Zhang D. 2021. Clinical epidemiology, risk factors, and control strategies of *Klebsiella pneumoniae* infection. Front Microbiol 12:750662. doi:10.3389/fmicb.2021.750662 34992583 PMC8724557

[4] Nusrat AA. 2025. Study on bacteriological profile and antimicrobial susceptibility pattern of Klebsiella pneumoniae and Escherichia coli from blood of suspect septicemia patient*.* BRAC University. Available from: https://dspace.bracu.ac.bd:8443/xmlui/handle/10361/26699

[5] Kamal, PhD AHM, Khatun W, Rahman A, Tomal GM. 2025. Microbial spectrum and antibiotic resistance patterns in elderly bacteremia cases: insights from the geriatric ICU of rajshahi medical college hospital. TAJ 38:22–30. doi:10.70818/taj.v38i04.0538

[6] Chen YC, Tsai IT, Lai CH, Lin KH, Hsu YC. 2024. Risk factors and outcomes of community-acquired carbapenem-resistant *Klebsiella pneumoniae* infection in elderly patients. Antibiotics 13:282. doi:10.3390/antibiotics13030282 38534717 PMC10967446

[7] Soraci L, Cherubini A, Paoletti L, Filippelli G, Luciani F, Laganà P, Gambuzza ME, Filicetti E, Corsonello A, Lattanzio F. 2023. Safety and tolerability of antimicrobial agents in the older patient. Drugs Aging 40:499–526. doi:10.1007/s40266-023-01019-3 36976501 PMC10043546

[8] Mangoni AA, Jackson SHD. 2004. Age‐related changes in pharmacokinetics and pharmacodynamics: basic principles and practical applications. Brit J Clinical Pharma 57:6–14. doi:10.1046/j.1365-2125.2003.02007.x PMC1884408 14678335

[9] D V, Ja B. 2015. Medication in the elderly - considerations and therapy prescription guidelines. Acta Medica Acad 44:159. doi:10.5644/ama2006-124.142 26702910

[10] Itani R, Khojah HMJ, Kibrit R, Raychouni H, Shuhaiber P, Dib C, Hassan M, Mukattash TL, El-Lakany A. 2024. Risk factors associated with multidrug-resistant *Klebsiella pneumoniae* infections: a multicenter observational study in lebanese hospitals. BMC Public Health 24:2958. doi:10.1186/s12889-024-20474-0 39449026 PMC11515809

[11] Aurilio C, Sansone P, Barbarisi M, Pota V, Giaccari LG, Coppolino F, Barbarisi A, Passavanti MB, Pace MC. 2022. Mechanisms of action of carbapenem resistance. Antibiotics 11:421. doi:10.3390/antibiotics11030421 35326884 PMC8944602

[12] Adeniyi Adefegha S. 2019. Antibiotics and drug pharmacology. Act Scie Pharma 3:43–49. doi:10.31080/ASPS.2019.03.0424

[13] Bush NG, Diez-Santos I, Abbott LR, Maxwell A. 2020. Quinolones: mechanism, lethality and their contributions to antibiotic resistance. Molecules 25:5662. doi:10.3390/molecules25235662 33271787 PMC7730664

[14] Li Y, Kumar S, Zhang L, Wu H, Wu H. 2023. Characteristics of antibiotic resistance mechanisms and genes of *Klebsiella pneumoniae*. Open Med 18:20230707. doi:10.1515/med-2023-0707 PMC10183727 37197355

[15] Birkegård AC, Halasa T, Græsbøll K, Clasen J, Folkesson A, Toft N. 2017. Association between selected antimicrobial resistance genes and antimicrobial exposure in danish pig farms. Sci Rep 7:9683. doi:10.1038/s41598-017-10092-9 28852034 PMC5575052

[16] Di Pilato V, Pollini S, Miriagou V, Rossolini GM, D’Andrea MM. 2024. Carbapenem-resistant *Klebsiella pneumoniae*: the role of plasmids in emergence, dissemination, and evolution of a major clinical challenge. Expert Rev Anti Infect Ther 22:25–43. doi:10.1080/14787210.2024.2305854 38236906

[17] Sukumaran A. 2023. Uncovering novel mechanisms of *Klebsiella pneumoniae* pathogenesis through mass spectrometry-based proteomics

[18] Rotondo C, Venditti C, Butera O, Dimartino V, Messina F, Properzi M, Caparrelli C, Antonelli V, D’Arezzo S, Selleri M, Nisii C, Fontana C. 2024. Molecular characterization of multidrug-resistant and hypervirulent new delhi metallo-beta-lactamase *Klebsiella pneumoniae* in Lazio, Italy: a five-year retrospective study. Antibiotics 13:1045. doi:10.3390/antibiotics13111045 39596739 PMC11590914

[19] Kochan TJ, Nozick SH, Medernach RL, Cheung BH, Gatesy SWM, Lebrun-Corbin M, Mitra SD, Khalatyan N, Krapp F, Qi C, Ozer EA, Hauser AR. 2022. Genomic surveillance for multidrug-resistant or hypervirulent *Klebsiella pneumoniae* among United States bloodstream isolates. BMC Infect Dis 22:603. doi:10.1186/s12879-022-07558-1 35799130 PMC9263067

[20] Yan JJ, Zheng PX, Wang MC, Tsai SH, Wang LR, Wu JJ. 2015. Allocation of *Klebsiella pneumoniae* bloodstream isolates into four distinct groups by ompK36 typing in a taiwanese university hospital. J Clin Microbiol 53:3256–3263. doi:10.1128/JCM.01152-15 26224840 PMC4572542

[21] Khan ER, Aung MS, Paul SK, Ahmed S, Haque N, Ahamed F, Sarkar SR, Roy S, Rahman MM, Mahmud MC, Hossain MA, Urushibara N, Kawaguchiya M, Sumi A, Kobayashi N. 2018. Prevalence and molecular epidemiology of clinical isolates of *Escherichia coli* and *Klebsiella pneumoniae* harboring extended-spectrum beta-lactamase and carbapenemase genes in Bangladesh. Microb Drug Resist 24:1568–1579. doi:10.1089/mdr.2018.0063 29958064

[22] Farzana R, Jones LS, Barratt A, Rahman MA, Sands K, Portal E, Boostrom I, Espina L, Pervin M, Uddin AKMN, Walsh TR. 2020. Emergence of mobile colistin resistance (*mcr-8*) in a highly successful *Klebsiella pneumoniae* sequence type 15 clone from clinical infections in Bangladesh. mSphere 5:e00023-20. doi:10.1128/mSphere.00023-20 32161143 PMC7067589

[23] Al-Amin S, Hassan MZ, Saif-Ur-Rahman KM, Chowdhury MAB, Morrison SD, Donevant SB, Chowdhury F. 2021. Pattern of antibiotic use for acute respiratory infections among out-patients in south asian region: protocol for a systematic review. Medicine 100:e22398. doi:10.1097/MD.0000000000022398 33530153 PMC7850708

[24] Gandra S, Alvarez-Uria G, Turner P, Joshi J, Limmathurotsakul D, Van Doorn HR. 2020. Antimicrobial resistance surveillance in low- and middle-income countries: progress and challenges in eight south asian and southeast asian countries. Clin Microbiol Rev 33:e00048-19. doi:10.1128/CMR.00048-19 32522747 PMC7289787

[25] Ahammed E, Islam MA, Akhter FB, Mim AA, Amin F, Nisa NA. 2025. Elderly vulnerability to infectious diseases in Bangladesh: an examination of comorbidities, hospital stay, and mortality. Asia Pac J Surg Adv 2:10–16. doi:10.70818/apjsa.2025.v02i01.014

[26] Chowdhury F, Mah-E-Muneer S, Bollinger S, Sharma A, Ahmed D, Hossain K, Hassan MZ, Rahman M, Vanderende D, Sen D, Mozumder P, Khan AA, Sarker M, Smith RM, Styczynski A, Luvsansharav U-O. 2023. Prevalence of colonization with antibiotic-resistant organisms in hospitalized and community individuals in Bangladesh, a phenotypic analysis: findings from the antibiotic resistance in communities and hospitals (ARCH) study. Clin Infect Dis 77:S118–S124. doi:10.1093/cid/ciad254 37406054 PMC10321696

[27] Mahbub A, Islam S, Chanda M, Akram A, Rahman MA. 2025. Observing the prevalence of antimicrobial resistance at a tertiary-level medical facility in Bangladesh. Eastern Med Coll J 10:57–64. doi:10.3329/emcj.v10i1.82571

[28] CLSI. 2026. AST news updates. Available from: https://clsi.org/resources/clsi-ast-news-updates/

[29] Tanni AA, Hasan MM, Sultana N, Ahmed W, Mannan A. 2021. Prevalence and molecular characterization of antibiotic resistance and associated genes in *Klebsiella pneumoniae* isolates: a clinical observational study in different hospitals in Chattogram, Bangladesh. PLoS One 16:e0257419. doi:10.1371/journal.pone.0257419 34506611 PMC8432802

[30] Samadi N, Kalani M, Azimi T, Hosainzadegan H, Hadi N. 2024. Phenotypic and molecular detection of carbapenemase-producing *Klebsiella pneumoniae* isolated from patients admitted to teaching hospitals in Shiraz, Iran. Jundishapur J Microbiol 16. doi:10.5812/jjm-142449

[31] Kulaç Ö, Başkan C, Koşar N, Balcı PÖ, Havuz SG, Sırıken B. 2024. *Klebsiella pneumoniae* clinical isolates: extended spectrum β-lactamase production, biofilm formation, and virulence factors. Biologia 79:3209–3217. doi:10.1007/s11756-024-01763-w

[32] Manojlovic Z, Wlodarczyk J, Okitsu C, Jin Y, Van Den Berg D, Lieber MR, Hsieh C-L. 2023. Construction of high coverage whole-genome sequencing libraries from single colon crypts without DNA extraction or whole-genome amplification. BMC Res Notes 16:66. doi:10.1186/s13104-023-06333-y 37106434 PMC10142246

[33] Kwak SM, Jeong JH, Oh JY, Chae JC. 2024. Complete genome sequence of multidrug-resistant *Enterococcus faecalis* strain CF3A-2134 isolated from feces of a pig. Microbiol Soc Korea 60:44–46. doi:10.7845/kjm.2024.4004

[34] Brown J, Pirrung M, McCue LA. 2017. FQC dashboard: integrates FastQC results into a web-based, interactive, and extensible FASTQ quality control tool. Bioinformatics 33:3137–3139. doi:10.1093/bioinformatics/btx373 28605449 PMC5870778

[35] Marino A. 2021. iSwap: a bioinformatics pipeline for index switching in illumina sequencing platforms. Available from: https://boa.unimib.it/handle/10281/314918

[36] Gurevich A, Saveliev V, Vyahhi N, Tesler G. 2013. QUAST: quality assessment tool for genome assemblies. Bioinformatics 29:1072–1075. doi:10.1093/bioinformatics/btt086 23422339 PMC3624806

[37] Prjibelski A, Antipov D, Meleshko D, Lapidus A, Korobeynikov A. 2020. Using SPAdes de novo assembler. Curr Protoc Bioinforma 70:e102. doi:10.1002/cpbi.102 32559359

[38] Tanni AA, Sharmen F, Chakma K, Yasmin F, Akash AS, Akash M, Riana SH, Afrin S, Ferdous J, Sultana N, Biswas SK, Islam SMR, Mannan A. 2024. Whole-genome sequencing of *Klebsiella pneumoniae* isolated from clinical specimens in Chattogram, Bangladesh. Edited by AO Hudson. Microbiol Resour Announc 13:e0044224. doi:10.1128/mra.00442-24 38940528 PMC11256831

[39] Lam MMC, Wick RR, Watts SC, Cerdeira LT, Wyres KL, Holt KE. 2021. A genomic surveillance framework and genotyping tool for *Klebsiella pneumoniae* and its related species complex. Nat Commun 12:4188. doi:10.1038/s41467-021-24448-3 34234121 PMC8263825

[40] Vieira A de A, Piccoli BC, Y Castro TR, Casarin BC, Tessele LF, Martins RCR, Schwarzbold AV, Trindade P de A. 2023. Pipeline validation for the identification of antimicrobial-resistant genes in carbapenem-resistant *Klebsiella pneumoniae*. Sci Rep 13:15189. doi:10.1038/s41598-023-42154-6 37709838 PMC10502106

[41] Overbeek R, Olson R, Pusch GD, Olsen GJ, Davis JJ, Disz T, Edwards RA, Gerdes S, Parrello B, Shukla M, Vonstein V, Wattam AR, Xia F, Stevens R. 2014. The SEED and the rapid annotation of microbial genomes using subsystems technology (RAST). Nucleic Acids Res 42:D206–14. doi:10.1093/nar/gkt1226 24293654 PMC3965101

[42] Sands K, Carvalho MJ, Portal E, Thomson K, Dyer C, Akpulu C, Andrews R, Ferreira A, Gillespie D, Hender T, et al.. 2021. Characterization of antimicrobial-resistant Gram-negative bacteria that cause neonatal sepsis in seven low- and middle-income countries. Nat Microbiol 6:512–523. doi:10.1038/s41564-021-00870-7 33782558 PMC8007471

[43] Kawser Z, Sridhar S, Kar S, Habib T, Mukta SA, Azad K, Hasan N, Kulsum U, Siddik AB, Rahman S, Tanni NN, Nesa M, Earl AM, Worby CJ, Turbett SE, Shamsuzzaman SM, Harris JB, Qadri F, LaRocque RC. 2025. Clinical and genomic characterization of *Klebsiella pneumoniae* infections in Dhaka, Bangladesh. J Glob Antimicrob Resist 41:52–58. doi:10.1016/j.jgar.2024.12.016 39725322 PMC12067484

[44] Aldali JA. 2025. *Klebsiella pneumoniae:* a multidrug-resistant pathogen, has emerged in Saudi Arabia. Front Microbiol 16:1689974. doi:10.3389/fmicb.2025.1689974 41140410 PMC12549642

[45] Zhong X shan, Li Y zhi, Ge J, Xiao G, Mo Y, Wen Y qi, Liu J-P, Xiong Y-Q, Qiu M, Huo S-T, Cheng M-J, Chen Q. 2020. Comparisons of microbiological characteristics and antibiotic resistance of *Klebsiella pneumoniae* isolates from urban rodents, shrews, and healthy people. BMC Microbiol 20:12. doi:10.1186/s12866-020-1702-5 31937244 PMC6961239

[46] Rahman MH, Akther S, Ahmed S, Shahadat MN, Munsi MN, Siddique AB. 2025. Epidemiological factors associated with the prevalence of mobile genetic elements, and antimicrobial resistance patterns in *Klebsiella pneumoniae* of farm environments in Bangladesh. BMC Med Genomics 18:114. doi:10.1186/s12920-025-02181-w 40619416 PMC12232692

[47] Prestinaci F, Pezzotti P, Pantosti A. 2015. Antimicrobial resistance: a global multifaceted phenomenon. Pathog Glob Health 109:309–318. doi:10.1179/2047773215Y.0000000030 26343252 PMC4768623

[48] Li L, Huang L, Liu X, Ye Y, Sai F, Huang H. 2023. Intensive care unit-acquired pneumonia caused by *Klebsiella pneumoniae* in China: risk factors and prediction model of mortality. Medicine 102:e33269. doi:10.1097/MD.0000000000033269 36961194 PMC10035998

[49] Tanni AA, Sultana N, Ahmed W, Hasan MM, Hossain MS, Noyon SH, Hossain MM, Mannan A. 2022. Investigating antimicrobial resistance and ESBL producing gene in *Klebsiella* isolates among neonates and adolescents in southern Bangladesh. Can J Infect Dis Med Microbiol 2022:7071009. doi:10.1155/2022/7071009 36249592 PMC9553706

[50] Murray CJL, Ikuta KS, Sharara F, Swetschinski L, Robles Aguilar G, Gray A, Han C, Bisignano C, Rao P, Wool E, et al.. 2022. Global burden of bacterial antimicrobial resistance in 2019: a systematic analysis. The Lancet 399:629–655. doi:10.1016/S0140-6736(21)02724-0 PMC8841637 35065702

[51] Lee AC, Mullany LC, Koffi AK, Rafiqullah I, Khanam R, Folger LV, Rahman M, Mitra DK, Labrique A, Christian P, Uddin J, Ahmed P, Ahmed S, Mahmud A, DasGupta SK, Begum N, Quaiyum MA, Saha SK, Baqui AH. 2020. Urinary tract infections in pregnancy in a rural population of Bangladesh: population-based prevalence, risk factors, etiology, and antibiotic resistance. BMC Pregnancy Childbirth 20:1. doi:10.1186/s12884-019-2665-0 PMC6938613 31892316

[52] Murad M, Poran GMS, Baidya K, Ahsan N. 2025. Genomic characterization of pan‐drug resistant *Klebsiella pneumoniae* KPNW isolated from UTI patient in Bangladesh. Microbiologyopen 14:e70064. doi:10.1002/mbo3.70064 41025610 PMC12481437

[53] Moniruzzaman M, Hussain MT, Ali S, Hossain M, Hossain MS, Alam MAU, Galib FC, Islam MT, Paul P, Islam MS, Siddiqee MH, Mondal D, Parveen S, Mahmud ZH. 2023. Multidrug-resistant *Escherichia coli* isolated from patients and surrounding hospital environments in Bangladesh: a molecular approach for the determination of pathogenicity and resistance. Heliyon 9:e22109. doi:10.1016/j.heliyon.2023.e22109 38027708 PMC10679508

[54] Mondal AH, Siddiqui MT, Sultan I, Haq QMohdR. 2019. Prevalence and diversity of bla TEM, bla SHV and bla CTX-M variants among multidrug resistant *Klebsiella* spp. from an urban riverine environment in India. Int J Environ Health Res 29:117–129. doi:10.1080/09603123.2018.1515425 30185065

[55] Robledo IE, Aquino EE, Vázquez GJ. 2011. Detection of the KPC gene in *Escherichia coli*, *Klebsiella pneumoniae*, *Pseudomonas aeruginosa*, and *Acinetobacter baumannii* during a PCR-based nosocomial surveillance study in Puerto Rico. Antimicrob Agents Chemother 55:2968–2970. doi:10.1128/AAC.01633-10 21444702 PMC3101463

[56] Haudiquet M, Buffet A, Rendueles O, Rocha EP. 2021. Interplay between the cell envelope and mobile genetic elements shapes gene flow in populations of the nosocomial pathogen *Klebsiella pneumoniae*. PLoS Biol 19:e3001276. doi:10.1371/journal.pbio.3001276 34228700 PMC8259999

[57] Domingues RB, Kuster GW, Onuki de Castro FL, Souza VA, Levi JE, Pannuti CS. 2006. Headache features in patients with dengue virus infection. Cephalalgia 26:879–882. doi:10.1111/j.1468-2982.2006.01100.x 16776706

[58] Davies YM, Cunha MPV, Dropa M, Lincopan N, Gomes VTM, Moreno LZ, Sato MIZ, Moreno AM, Knöbl T. 2022. Pandemic clones of CTX-M-15 producing *Klebsiella pneumoniae* ST15, ST147, and ST307 in companion parrots. Microorganisms 10:1412. doi:10.3390/microorganisms10071412 35889131 PMC9320316

[59] Zhen X, Lundborg CS, Zhang M, Sun X, Li Y, Hu X, Gu S, Gu Y, Wei J, Dong H. 2020. Clinical and economic impact of methicillin-resistant *Staphylococcus aureus*: a multicentre study in China. Sci Rep 10:3900. doi:10.1038/s41598-020-60825-6 32127606 PMC7054446

[60] Malik B, Bhattacharyya S. 2019. Antibiotic drug-resistance as a complex system driven by socio-economic growth and antibiotic misuse. Sci Rep 9:9788. doi:10.1038/s41598-019-46078-y 31278344 PMC6611849

[61] Poudel AN, Zhu S, Cooper N, Little P, Tarrant C, Hickman M, Yao G. 2023. The economic burden of antibiotic resistance: a systematic review and meta-analysis. PLoS One 18:e0285170. doi:10.1371/journal.pone.0285170 37155660 PMC10166566

[62] Gandra S, Barter DM, Laxminarayan R. 2014. Economic burden of antibiotic resistance: how much do we really know? Clin Microbiol Infect 20:973–980. doi:10.1111/1469-0691.12798 25273968

